# Physicians' Attitudes to Clinical Pain Management and Education: Survey from a Middle Eastern Country

**DOI:** 10.1155/2016/1358593

**Published:** 2016-03-29

**Authors:** Soumana C. Nasser, Jeanette G. Nassif, Aline Hanna Saad

**Affiliations:** Department of Pharmacy Practice, School of Pharmacy, Lebanese American University, P.O. Box 36 (S23), Byblos 961, Lebanon

## Abstract

Despite promising initiatives to advance the practice of pain management in Middle Eastern countries, their pain care lags behind developed countries. The objectives of this study are to evaluate physicians' assessment of their own competency in pain management, to assess physicians' practice related to pain management, and to identify physician-related barriers to effective pain control. A cross-sectional survey was conducted in 3 teaching medical centers in Lebanon targeting the above-mentioned outcomes and assessing the impact of physicians' years in practice on the studied end-points. A total of 69 physicians were surveyed. Fifty-seven percent reported “very good to excellent” pain management skills; only 25% of them described the need for continuing professional development. When treating patients with pain, 52% of physicians refer to updated international guidelines, whereas 43% rely on their own judgment. Physicians were more likely to consult with another physician (65%) rather than a pharmacist (12%) when treating patients with pain. Fear of adverse effects of analgesics was the most commonly reported barrier (45%) to pain control among physicians from different career stages. Based on these survey findings, national pain management and practice policies are needed to optimize this area of deficiency in patient care.

## 1. Introduction 

Pain is a universal health problem with an estimated worldwide prevalence of 20% [1]. Undertreatment of pain remains a global concern [2] as documented by studies suggesting that 30% to 40% of early stage cancer patients and 70% to 80% of those receiving therapy or with advanced disease have unsatisfactory pain control [3]. Canadian studies also document inadequate pain management in emergency care [4], critical care [5], palliative care [6], and chronic pain [7]. Inadequately treated pain adversely affects patients' quality of life, physical and psychological wellbeing, sleep patterns, and activities of daily living [2]. Despite guidelines developed to improve patient pain control, clinicians often fail to follow them perhaps due to their insufficient knowledge of pain management and inaccurate pain assessment [3, 8]. Other listed reasons included patient- and system-related barriers [8–10].

Pain relief for palliative care in the Middle East is inadequate. Effective reform and development of pain management strategies are required [11]. As many patients in these countries have relatively advanced diseases and low chances for cure, the need for better pain management and palliative care services is urgent. Barriers to implementation include insufficient training of health care workers in this field, lack of health policies encouraging this service, restricted accessibility to and/or availability of opioids due to regulatory obstacles, and lack of knowledge or false beliefs related to opioids including abuse concern [12, 13]. The extent of these deficiencies varies from one country to another due to diversity in social and health indicators such as population size, economy, and total health care expenditure [14]. In addition, the Middle Eastern countries, including Lebanon, encompass a diversity of religions, cultures, and traditions. Patients have different perceptions related to pain, disease, and death. This imposes an additional barrier for health care professionals [15]. Daher (2011) identified potential impediments to adequate pain control in Lebanon including national policy (restrictive laws and regulations that govern the medical use of opioids), barriers in the provision of health services, and patient-related concerns. In this same review, the author endorsed several corrective measures at all levels to ameliorate pain management services in the country [14]. In parallel, the National Committee for Pain Control and Palliative Care (NCPCPC) was developed in 2011 under the auspice of the Lebanese Ministry of Public Health. The primary goals of this committee are to set standards for palliative care services and improvement of pain management and improve pain management and palliative care in Lebanon. These standards emphasize that physicians shall acquire the necessary training and competency in this field, which could be realized primarily by integrating pain and palliative care in undergraduate medical and nursing curricula [14, 16]. Despite these promising initiatives, their implementation remains uncertain. Understanding physicians' contribution as crucial providers of pain management needs further investigation. Thus, the aim of this study is (1) to evaluate physicians' assessments of their own competency in pain management, (2) to assess physicians' practices related to pain management, (3) to identify physician-related barriers to effective pain control, and (4) to investigate the impact of physicians' years in practice on these variables.

## 2. Methods

### 2.1. Study Design

A cross-sectional survey was conducted between November 2012 and May 2013 in 3 teaching medical centers in Lebanon located in different geographic areas. Independent variables of interest included physicians' years in practice while dependent variables were physicians' competency, approach to practice, and identified barriers related to pain management.

### 2.2. Participants' Enrollment

The survey was designed to reach out to 25 physicians per center for a total of 75 targeted participants. Number of surveyed participants per service varied depending on service size in each center. This number ranged from 2 to 10 participants from each of the following services: anesthesiology, intensive care, internal medicine, general and pediatric surgery, orthopedics, oncology, and obstetrics/gynecology. An IRB approval for this study was granted by the Lebanese American University Institutional Review Board.

### 2.3. Data Collection

A survey was developed by the investigators in English and adapted to the medical practice in Lebanese hospitals. The survey was then pilot tested, before administration, to ensure validity and clarity of included questions. Physicians were identified with the help of the central pharmacy department and then contacted for their willingness to fill out the questionnaire. A cover letter was provided to participants, detailing the purpose of the survey, the estimated time to complete the questionnaire, and reassuring confidentiality. Participating physicians were asked to voluntarily and anonymously fill this survey which included 14 questions to capture (1) physicians' assessments of their own competency in pain management based on their training and practice; (2) physicians' approach to pain management; and (3) their perceived barriers to effective pain management. Third year pharmacy students from the Lebanese American University School of Pharmacy were trained by their clinical preceptor to assist physicians in completing the surveys. Following completion, surveys were sealed and then submitted to the primary investigator.

The questionnaire explored participants' views on the need for formal pain management training as well as the types of references they use as resources. It also addressed how frequently participants cared for patients with pain, what pain scale they used, whether they consulted with other health care providers, and how they monitored posttreatment changes in pain intensity.

We contacted the 7 schools of medicine in the country to describe their medical curricula on pain management core competencies. We also approached the Lebanese Order of Physicians to identify requirements for pain competencies in medical schools' curricula, for offering or requiring pain management continuing education credits, and for any other pain management practice requisites. It is worth noting that the Lebanese Order of Physicians is highly engaged in organizing and coordinating national medical conferences inviting local and international speakers to keep physicians and medical students abreast of the latest medical practices. We also sought the Ministry of Public Health's input and initiatives on pain education and practice.

### 2.4. Statistical Analysis

Completed surveys were entered into an excel sheet using a coding system, and then data were analyzed using the SPSS version 18 software. When physicians were asked to rank their competency in pain management, the adopted scale was a range of 0 to 10 with below average corresponding to a score of less than 4, average to good corresponding to a score of 4 to 6, and very good to excellent corresponding to a score of 7 to 10. In order to facilitate data interpretation, physicians were grouped into three categories based on their years in practice defined as early career (less than or equal to 5 years in practice), mid-career (6 to 10 years), and advanced career (more than or equal to 11 years). A comparison was then conducted based on these three categories in relation to the study primary objectives. Variables were summarized using frequencies and percentages. The association between categorical variables was evaluated using Pearson *χ* ^2^ test or Fisher's exact test where the expected cell count was less than 5.* p* values below 0.05 were considered to be statistically significant.

## 3. Results

A total of 69 physicians (92% of physicians contacted) responded to the survey from 3 teaching medical centers in Lebanon. Their specialties included internal medicine 43.46%, anesthesia 24.64%, surgery 11.6%, and others. More than half of the surveyed physicians were classified as early career (55%) compared to 11.6% as mid-career and 33.3% as advanced career physicians (Table 1).

### 3.1. Physicians' Assessment of Their Own Competency in Pain Management Based on Years in Practice

Approximately 57% of physicians reported “very good to excellent” pain management skills. The lowest self-ratings were observed among early career physicians (45%). Only 25% of surveyed physicians expressed the need for continuing professional development (CPD) to improve their knowledge in pain management. One-third of early career physicians (32%) suggested more CPD. However the correlation between physicians' self-ratings of their competency in pain management and the need for CPD were not significantly associated with their years in practice (*p* = 0.084 and *p* = 0.569). About half of the physicians reported recent exposure to formal training, workshops, and updated courses on pain management. The lowest rate was seen in early career physicians (34%) ant the highest rate in advanced career physicians (78%). This indicates that physicians' exposure to CPD can be influenced by their years in practice (*p* = 0.003).

Half (52%) of respondents refer to updated international pain management guidelines as their primary resources; 43% rely on their own judgment when treating patients with pain, and 26% utilize more than one resource. The use of specific resources was not significantly related to physicians' years in practice (Table 2).

### 3.2. Physicians' Approach to Pain Management Based on Years in Practice

Around 93% of surveyed physicians rounded daily on patients experiencing pain in the hospital. Eighty-seven percent assessed their patients' pain routinely with no significant differences among career stages. Verbal assessments were most commonly used (72.5%) followed by the use of a visual analogue scale (29%). Advanced career physicians were more likely to use a visual analogue scale than the early career cohort (15.79%, *p* = 0.020). When physicians sought consultation on pain management, most of them referred to another physician (65%) or a nurse (42%) than to a pharmacist (12%). The early and mid-career physicians were more likely to consult with another health care professional compared to those in their advanced career (*p* = 0.025). Early career physicians were least likely to consult with a pharmacist (2.63%) compared to those in mid- (25%) and advanced career (21.74%) (*p* = 0.020). Around 70% of surveyed physicians used pain scores as a method to document pain improvement in patients after interventions, which is the case mainly with early career physicians (84.2%, *p* = 0.010) compared to physicians with more years in practice. Mid- to advanced career practitioners were more likely to use the patient satisfaction rating scale (62.5% of mid-career and 65.22% of advanced career physicians) (*p* = 0.045) when compared to early career practitioners and shortening length of hospital stay was more often adopted by mid-career physicians (62.5%, *p* = 0.005) (Table 3).

### 3.3. Barriers to Adequate Pain Management Based on Physician's Years in Practice

Surveyed physicians reported inadequate pain control to be related to the following issues:

(1) fear of adverse effects of analgesics, especially narcotics (45%), with no statistically significant difference between different career stages (*p* = 0.485),
(2) poor knowledge of pain management reported by 42% of physicians, with a statistically significant difference among various career stages, with the highest rate reported by mid-career physicians (100%) compared to others (*p* < 0.001),
(3) lack of response to conventional analgesics identified as a barrier by 30% of surveyed physicians, as was terminal illness (25%) with no statistically significant differences reported across years in practice (*p* = 0.564 and *p* = 0.122, resp.),
(4) forty-two percent of physicians reporting the barriers to appropriate management to be multifactorial (Table 4).

### 3.4. Pain Education in Medical Schools Curricula

We reviewed the websites of Schools of Medicine (SOM) in the country for their curricula content of pain education; little if any information was available and no data were published in the literature. We contacted all seven SOMs in Lebanon and asked about their provision of pain management education in their curricula. Five schools responded (see Table 5). All five schools incorporate pain education in their curricula and four of them reported addressing all types of pain throughout their programs (acute, chronic, postoperative, complex cancer, and AIDS related). Medical students are exposed to pain competencies through various courses in a fragmented way that span over their medical years (mainly during medical years 1 and 2). One school offers their medical students pain education through simulation activities as part of the clinical skills program (objective structured clinical simulation or OSCE). There were no compulsory pain management rotations, but one school reported rotations in pain research and pain management clinics as available options for the medical interns. The related students learning objectives were mostly limited and covered the basics of pain education as detailed in Table 5.

## 4. Discussion

This cross-sectional study assessed physicians' perceptions of their pain management skills, approach to pain management, and barriers to adequate pain control based on their years in practice. More than half of the surveyed physicians felt confident with their pain management skills and rated them as very good to excellent. Such confidence was lowest with early career physicians and could be explained by lack of experience compared to mid- and advanced career colleagues. Our results are slightly more positive than published literature on cancer pain whereby a survey administered to 4618 British Columbian physicians to assess their cancer pain knowledge and attitudes showed that 49% of physicians felt that their knowledge of cancer pain management was adequate while 35.5% did not, and 10.2% did not know [17]. Additionally, a survey of 91 interns, residents, and attending physicians assessing their confidence in pain management also showed that attending physicians had the highest confidence in pain management skills followed by senior residents and lastly by interns [18]. However, this relatively high confidence in pain knowledge could reflect that many physicians do not know what they should know, that is, evidence-based pain management strategies.

In addition, in countries as Lebanon, the physician makes all treatment-related decisions. The dominant cultural placement of physicians in most Middle Eastern countries could also explain their overestimation of their competency and skills. Pain education throughout the medical school curricula is not yet well-integrated, systematic, or comprehensive. Our findings of pain education in Lebanese medical schools curricula are in line with the available literature describing pain education for North American medical students and reporting it as variable and suboptimal [19]. In an effort to overcome these deficiencies worldwide, two organizations outlined core pain management competencies for various health care professional curricula [20, 21]. Accordingly, the available information on pain education in the country could not really explain our findings but a more comprehensive national study assessing the pain education of health care professionals is warranted. Such a study would provide the nation with a blueprint for an all-inclusive and structured pain education curriculum. This reinforces Daher's (2011) statement that pain education in Lebanon must be integrated in a systematic and multidisciplinary approach in all health care school curricula [14].

In 2013, the Lebanese Ministry of Public Health, through the National Committee for Pain Control and Palliative Care [16], recommended training to all physician and nursing staff in pain assessment, documentation, and management. Although two-thirds of surveyed physicians in this study did not feel the necessity to enhance their knowledge through further formal training, 50% of them have recently participated in workshops, courses, or formal training sessions on pain management. Advanced career physicians reported statistically significant higher rates of recent exposure to formal training, workshops, and updated courses on pain management. A national study on palliative care, however, reveals that 99% of surveyed practicing physicians and nurses required CPD. The same study revealed that only 16.7% of nurses and 12.3% of physicians received education in palliative care [22]. Such continuing education activities are currently not mandated by the Lebanese Order of Physicians for continued licensure. Programs are made available to health care professionals through various nongovernmental organizations such as the Lebanese Society for the Study of Pain (LSSP, a chapter of the International Association for the Study of Pain IASP) and the Pain Relief and Palliative Care Group (PR & PCG) that is under the auspices of the Lebanese Cancer Society [11]. Reviewed international studies on pain management knowledge and attitudes for health care professionals recommended continuing professional education, pain management skills development, and protocol development for the standardization of care [23].

In the surveyed academic centers, hospital-based pain management protocols exist for assisting physicians in their patient care. Our study revealed that the majority of physicians rely on international guidelines or their own judgment when it comes to resources on pain management. This finding is in line with a recent study in the country describing the status of compliance with pain management guidelines and emphasizing the need for a national effort to standardize and structure pain care across hospitals [24]. Institutions could disseminate their hospital-based guidelines through grand rounds, workshops, seminars, and references provision to increase physicians' compliance with guidelines and ensure consistency in management. Lebanese physicians are mostly counting on their personal knowledge and experience to deliver care rather than on standardized institutional or national policies and procedures. This phenomenon is not restricted to Lebanon. In a survey of 91 American physicians, only 23% of respondents were aware of their institution's pain management guidelines. This study emphasized the need for innovative educational initiatives to overcome this challenge [18].

Our study results showed inadequate implementation of an interdisciplinary approach in pain management and pharmacist engagement was poor as compared to other health care providers. When needing assistance in decision making on pain management, physicians mostly consulted with other physicians; nurses were sought next; and pharmacists ranked last. A study by Abu-Saad Huijer and Dimassi addressed the issue of multidisciplinary involvement in palliative care provision in Lebanon. The results showed that around 90% of palliative care physicians involved nurses in their decision-making process. However, the study did not include pharmacists [22]. Our findings reflect the current limited clinical role of pharmacists in Lebanon and their dispensing driven functions rather than patient-centered ones. Currently, pharmacists are slowly but steadily transforming the culture of the profession in the country towards drug experts and patient care providers through a national coalition force that is committed to transform the role of pharmacists as health care professionals trained in patient-centered care [25, 26]. Pharmacists can play an instrumental role in implementing pain management guidelines; safeguarding adequate opioid prescribing; and providing educational resources to patients and other health care providers [27].

The barriers identified by Lebanese physicians are similar to those found in other countries, with fear of adverse effects of analgesics, especially opioids (45%) and poor knowledge of pain management (42%) ranking at the top. International studies from Canada [28], France [29], and the United States of America [30] have also highlighted the role of insufficient physicians' knowledge in pain management as a contributing factor to ineffective pain control. Physicians' attitudes and educational barriers and the limitations of available analgesics and misconceptions surrounding their use [31] are among the most commonly cited barriers to adequate pain management [2]. The most important perceived barriers to optimal pain control amongst cancer pain patients were physicians' inadequate knowledge about opioid analgesics along with fear of tolerance, drug addiction, and side effects [32]. Opioids are available in all Middle Eastern countries; however, they can only be prescribed by oncologists in half of these countries, with the duration of the prescription varying from 1 week to 1 month [14].

In Lebanon, and until 2009, only cancer patients had access to opioids. National legislative changes have emphasized the right of every patient to appropriate pain management and access to opioids [11]. Oncologists, anesthesiologists, family physicians, and hematologists can now prescribe opioids [15]. This is a step in the right direction, but relatively limited number of opioids are available in Lebanon because of high cost, difficulty in the prescribing process through the Ministry of Public Health, poor health care professional training, and fear of opioid abuse [11]. Opioid consumption, an indicator of the quality and quantity of palliative care provided, is still low in Middle Eastern countries compared with the developed world. In fact, morphine consumption in Lebanon is higher than that in other Middle Eastern countries but still lags behind most North American and European countries [14].

A number of parameters assessed in this study (CPD, pain assessment scales, consultation with other health care professionals, and pain monitoring) were influenced by physicians' years in practice. Our findings complement reports in the literature detailing the impact of health care professionals' characteristics on pain management decisions while providing supplementary insight on the years in practice effect on pain management. A recent study conducted by Bartley et al. revealed statistically significant influences of practitioners' gender, race, age, and duration of professional experience on pain management decisions [33]. Tarigopula et al. [34] also examined the characteristics of health care professionals in an intensive care unit (ICU) setting and their impact on pain decision making. They concluded that race, age, level of education, and medical subspecialty were significant parameters impacting the health care professionals' perceptions of pain management and decision making [34].

Our study investigated the current status of pain management in Lebanon from physicians' perspective after launching several initiatives and reform strategies in recent years to improve the provision of pain management in the country. Three teaching hospitals were involved in this study allowing multicenter input. Despite the small number of physicians surveyed, the response rate was high. This study can serve as a snapshot for larger studies where physician's specialty, besides years in practice, can be assessed as an independent variable affecting pain management practice. Our study may help build the national database on pain management from the perspectives of health care providers, as its results will be shared with the NCPCPC and the Lebanese Ministry of Public Health. The study will help responsible authorities to grasp information on the current status of physicians' attitudes towards pain management. Thus, further observational reports and consequent actions can be taken to develop and better implement the recommended initiatives in the field. Future endeavors should focus on patients' perspectives of pain management, health care professionals' pain education, health care providers actual pain knowledge (e.g., the Know-Pain 50 score), and implementation of national pain management protocols.

## 5. Conclusion

Our study assessed physicians' perceptions of their own knowledge and approach to pain management, as well as the barriers to the provision of pain management in Lebanon. Primary results indicate that despite the recent initiatives adopted by nongovernmental professional bodies towards improving palliative care and pain management services, additional improvements are required. Our nation is strengthening its efforts to address its deficiencies in pain management by incorporating its newly established policies and procedures. A systematic and comprehensive educational approach for integrating pain and palliative care into the medical, nursing, and pharmacy school curricula is a priority. National policies should support the training of all health care providers in improving their knowledge of pain management and formulating hospital-based protocols along with a follow-up strategy to evaluate outcomes of any implemented changes. These policies should also recognize the importance of an interdisciplinary approach by engaging pharmacists and nurses in treating patients with pain. No patient in Lebanon should be left to suffer pain.

## Figures and Tables

**Table 1:** Characteristics of participants.

Characteristics of participants	Number of surveyed physicians (*N* = 69)	Percent (%)
Department		
Internal medicine	30	43.46
Anesthesia	17	24.64
Surgery	8	11.60
Oncology	5	7.25
Orthopedics	5	7.25
Pediatrics surgery	2	2.90
Obstetrics/gynecology	2	2.90
Physicians' years in practice		
Early career (≤5 years)	38	55.07
Mid-career (5–10 years)	8	11.60
Late career (≥11 years)	23	33.33

**Table 2:** Physicians' assessment of their own competency in pain management based on years in practice.

	Total number of physicians	Years in practice			*p* value
	*N* = 69	Early career (≤5 years) *N* = 38	Mid-career (6–10 years) *N* = 8	Advanced career (≥11 years) *N* = 23	
*Rating physicians' expertise in pain management*					
Average to good	30 (43.48%)	21 (55.26%)	2 (25.0%)	7 (30.43%)	
Very good to excellent	39 (56.52%)	17 (44.74%)	6 (75.0%)	16 (69.57%)	0.084
*Necessity for a formal training to improve knowledge in pain management*					
Yes	17 (24.64%)	12 (31.58%)	1 (12.5%)	4 (17.39%)	
No	46 (66.67%)	23 (60.53%)	7 (87.5%)	16 (69.57%)	
No answer	6 (8.69%)	3 (7.89%)	0 (0%)	3 (13.04%)	0.569
*Any recent formal training/workshop/course on pain management*					
Yes	35 (50.72%)	13 (34.21%)	4 (50.0%)	18 (78.26%)	
No	34 (49.28%)	25 (65.79%)	4 (50.0%)	5 (21.74%)	0.003
*Pain management reference used*					
Hospital protocol on pain management	15 (21.74%)	11 (28.95%)	2 (25.0%)	2 (8.70%)	0.173
Other published hospital protocols	6 (8.70%)	2 (5.26%)	2 (25.0%)	2 (8.70%)	0.188
Physician's own judgment	30 (43.48%)	15 (39.47%)	4 (50.0%)	11 (47.83%)	0.731
Updated international pain management guidelines	36 (52.17%)	15 (39.47%)	5 (62.5%)	16 (69.57%)	0.067
More than one answer	18 (26.09%)				

**Table 3:** Physicians' approach to pain management based on years in practice.

	Total number of physicians	Years in practice			*p* value
	*N* = 69	Early career (≤5 years) *N* = 38	Mid-career (6–10 years) *N* = 8	Advanced career (≥11 years) *N* = 23	
*Making hospital rounds on patients in pain everyday*	64 (92.75%)	36 (94.74%)	7 (87.50%)	21 (91.30%)	0.372
*Routine patient's pain assessment*	60 (86.96%)	33 (86.84%)	5 (62.50%)	22 (95.65%)	0.062
*Method for pain assessment*					
Visual analogue scale	20 (29%)	6 (15.79%)	3 (37.5%)	11 (47.83%)	0.020
Numerical scale	12 (17.4%)	8 (21.05%)	0 (0.00%)	4 (17.39%)	0.533
Verbal communication	50 (72.5%)	27 (71.05%)	6 (75.0%)	17 (73.91%)	1.000
*Consulting with other health care providers for pain management*					
Nurse	29 (42.03%)	14 (36.84%)	4 (50.00%)	11 (47.83%)	0.610
Pharmacist	8 (11.59%)	1 (2.63%)	2 (25.00%)	5 (21.74%)	0.020
Physician	45 (65.22%)	28 (73.68%)	7 (87.50%)	10 (43.48%)	0.025
More than one answer	13 (18.84)				
*Measurement of pain performance improvement after intervention*					
Pain score	49 (71.01%)	32 (84.21%)	3 (37.50%)	14 (60.87%)	0.010
Improved patient satisfaction ratings	33 (47.83%)	13 (34.21%)	5 (62.50%)	15 (65.22%)	0.045
Shortened length of hospital stay	16 (23.19%)	4 (10.53%)	5 (62.50%)	7 (30.43%)	0.005
More than one answer	29 (42.03%)				

**Table 4:** Barriers to adequate pain management based on physicians' years in practice.

	Total number of physicians	Years in practice			*p* value
	*N* = 69	Early career (≤5 years) *N* = 38	Mid-career (6–10 years) *N* = 8	Advanced career (≥11 years) *N* = 23	
Pain is usually unresponsive to conventional analgesics	21 (30.43%)	13 (34.21%)	1 (12.50%)	7 (30.43%)	0.564
Terminal illness	17 (24.64%)	12 (31.58%)	0 (0.00%)	5 (21.74%)	0.122
Poor knowledge of pain management	29 (42.03%)	10 (26.32%)	8 (100.0%)	11 (47.83%)	<0.001
Fear of adverse effects of analgesics particularly respiratory depression and addiction to opioids	31 (44.93%)	17 (44.74%)	2 (25.00%)	12 (52.17%)	0.485
More than one answer	29 (42.03%)				

**Table 5:** Pain education in Lebanese medical schools curricula.

Questions	Medical school 1	Medical school 2	Medical school 3	Medical school 4	Medical school 5
Do you teach medical students pain assessment and management in your didactic curriculum?	Yes	Yes	Yes	Yes	Yes

During which academic year(s) do you deliver your didactic curriculum on pain assessment and management?	Medical years 1 and 2	Medical year 2	Medical years 1, 2, and 3	Medical year 2	Medical year 3

What types of pain are covered in the didactic curriculum?	Acute, chronic, postoperative, and/or cancer pain	Acute, chronic, postoperative, functional, and/or complex (cancer/AIDS pain)	Acute, chronic, postoperative, palliative, and cancer pain	No answer	All types of pain

Do you have a *separate course* dedicated to teaching your medical students on pain assessment and management?	No, it is taught within various courses	No answer	No, it is taught within various courses	No, it is taught within various courses	No, it is taught within various courses

Do you teach medical students on pain assessment and management in *simulation settings*?	Yes	No	No	No	No

Do you teach medical students on pain assessment and management in your *experiential* curriculum?	No	Yes	No	No	No

Do you have a separate *rotation* dedicated to pain assessment and management?	No	Yes	No	No	No

Kindly share student learning objectives that must be met on pain assessment and management in your curriculum	(i) Briefly review the mechanisms underlying pain sensation and describe clinical assessment of pain (ii) Discuss selection of pain medications based on pain severity and type (iii) Describe various modalities used to prevent and treat pain in different patient situations such as postoperative pain and cancer pain (iv) Discuss the indications and side effects of patient-controlled analgesia (v) Discuss the aims of pain management programs according to international guidelines (vi) Discuss alternative interventions for pain management	(i) What is pain? (ii) How is pain recognized? (iii) What are the neural substrates of pain? (iv) How does context influence pain? (v) How is pain relieved?	(i) Physiology (mechanism, different types) (ii) Surgery (traumatology, acute pain) (iii) Oncology (palliative care, cancer pain) (iv) Anesthesia (pain control and management)	No answer	(i) Be aware of the different type of pain (ii) Know the different scores to evaluate pain in adults, pediatric population, and elderly patients (iii) Medical management (classes of analgesics) (iv) Nonmedical treatment (v) Surgical techniques in selected patients

Does your affiliated hospital offer medical residents the option of a *residency program* in pain management?	No	No	Yes	No	No
